# Treatment of acute myocardial infarction in the sub-arctic region of Norway. Do we offer an equal quality of care?

**DOI:** 10.1080/22423982.2017.1391651

**Published:** 2017-10-26

**Authors:** J. Norum, A. Hovland, L. Balteskard, T. Trovik, B. Haug, F. H. Hansen, S. Alterskjær, P. Madsen, F. Olsen

**Affiliations:** ^a^ Department of Surgery, Finnmark Hospital, Hammerfest, Norway; ^b^ Department of Clinical Medicine, Faculty of Health Science, UiT - The Arctic University of Norway, Tromsø, Norway; ^c^ Department of Cardiology, Nordland Hospital, Bodø, Norway; ^d^ Centre for Clinical Documentation and Evaluation, Northern Norway Regional Health Authority trust, Tromsø, Norway; ^e^ Department of Cardiology, University Hospital of North Norway, Tromsø, Norway; ^f^ Department of Medicine, Helgeland Hospital, Sandnessjøen, Norway; ^g^ Northern Norway Regional Health Authority trust, Bodø, Norway; ^h^ National Air Ambulance Services of Norway, Bodø, Norway

**Keywords:** Acute myocardial infarction, Norway, incidence, service, air ambulance

## Abstract

Patients, relatives, healthcare workers and administrators are concerned about the quality of care offered. We aimed to explore the treatment of acute myocatrdial infarction (AMI) in Northern Norway, compare it with the national figures, and document whether there is an equal quality of care or not. The retrospective study included data on patients' treatment for AMI. The following sources were employed. The Norwegian Patient Registry, National Quality of Care Database, Norwegian Myocardial Infarction Registry and data from the National Air Ambulance Services of Norway. The period 2012-2014/15 was studied and the variables were: incidence of AMI, gender and age adjusted rates of AMI and revascularization (PCI, CABG) based on patient's place of living (according to hospital catchment area) and 30-day survival rate. The annual incidence of AMI was 9% higher in the northern region. Significant incidence variations (2.7–5.9 AMI/1000 inhabitants) between the hospitals' catchment areas were revealed. The 30-day survival rate varied between 85.1–92.1% between hospitals. The variation in revascularization/AMI rate was 0.72–1.54. Air amublance services' availability varied through the day. In conclusion, significant variations in the AMI rate and an unequal service within the region was revealed.

## Introduction

Appropriate interventions may decrease the disability and death rates due to acute myocardial infarction (AMI) [1–4]. Several countries have added AMI to the list of targets to be monitored and assessed at the national level [1–4]. In this monitoring, hospital performance has been compared based on quality of care measures [5–9]. During the last decade, the incidence of AMI and the case-fatal rate (CRF) has decreased in several countries, including Norway [10,11]. The improvements have been due to advances in invasive treatments and medical management, such as pre-hospital thrombolytic therapy and primary percutaneous coronary intervention (PCI). However, regional variations in the AMI and CFR and the causes thereof have been reported [12–14]. Disproportionate differences in the medical infrastructure, available in the main cities with PCI-centres versus in other areas (without direct access to a PCI centre), have been a focus of growing concern in Norway and other countries [15].

Norway comprises the western portion of the Scandinavian Peninsula, plus the island Jan Mayen and the archipelago of Svalbard. The country has four health regions. The northern region constitutes 45% of Norway’s land mass (Svalbard inclusive), but has only 9.4% (0.5 million) of the country’s population (5.2 million). Despite people being scattered within a substantial area (173,966 km^2^), they have been promised, by the Northern Norwegian Regional Health Authority (NNRHA) trust, a healthcare of equal quality within the whole region. To meet such expectations, the NNRHA trust runs 11 medical hospitals and has 12 available air ambulance resources (six fixed wing aircrafts, four ambulance helicopters, two search and rescue helicopters) scattered on the mainland [16,17]. The regional PCI-centre is located at the University hospital of North-Norway (UNN) in Tromsø. In this study, we aimed to explore whether all these resources offer patients suffering from AMI a similar quality of care within Northern Norway and compare the figures to the national ones.

## Materials and methods

### Data sources

**Figure 1.**
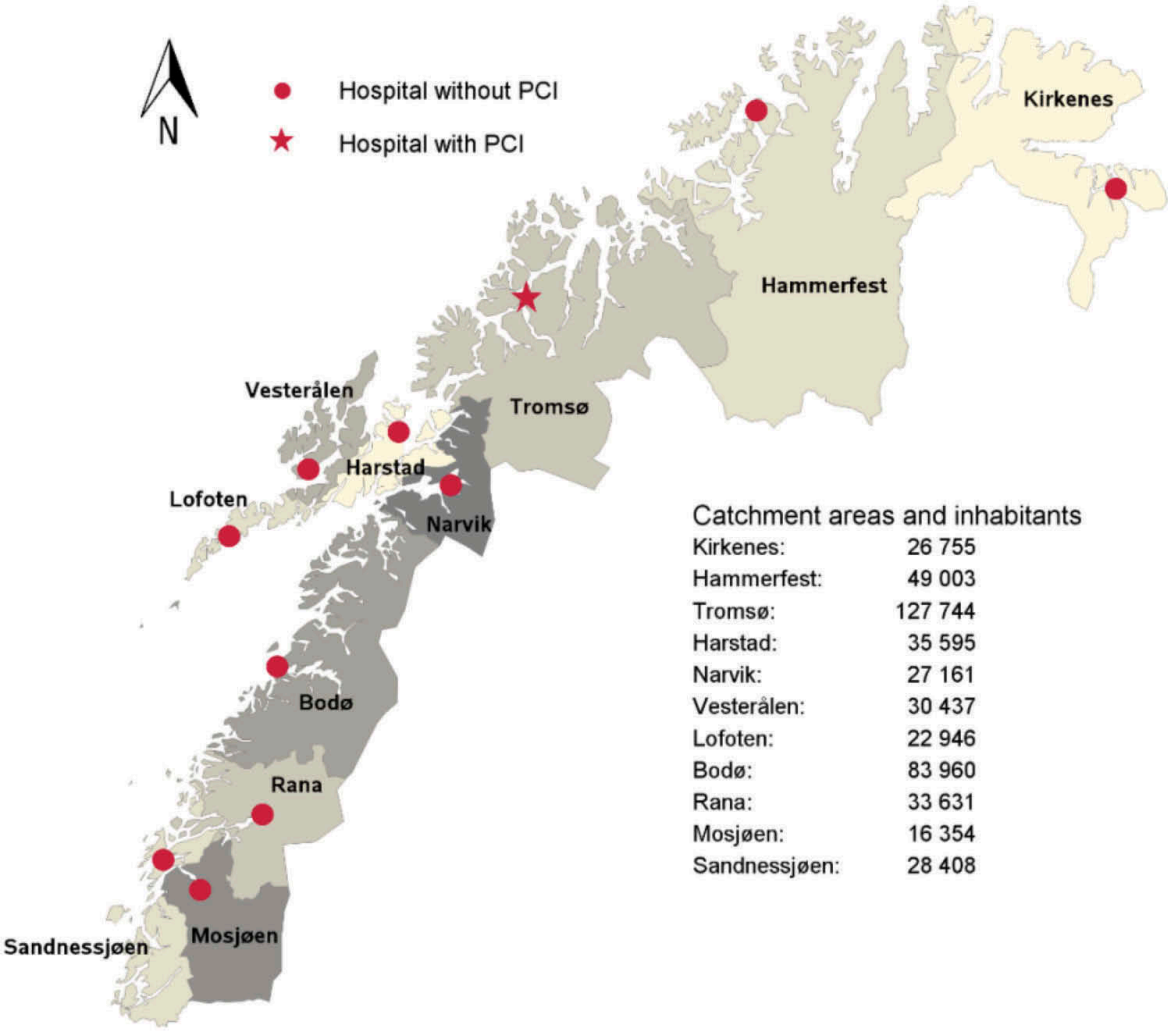
Locations and catchment areas of the 11 medical hospitals on the northern Norwegian mainland.

This study was undertaken to document the quality of care of AMI in Northern Norway. A retrospective design was used and the treatment of AMI within the hospitals’ catchment areas was analysed. The hospitals were Finnmark hospital Kirkenes, Finnmark hospital Hammerfest, University hospital of North-Norway Tromsø, University hospital of North-Norway Harstad, University hospital of North-Norway Narvik, Nordland hospital Vesterålen, Nordland hospital Lofoten, Nordland hospital Bodø, Helgeland hospital Rana, Helgeland hospital Sandnessjøen and Helgeland hospital Mosjøen. Locations and catchment areas are shown in Figure 1. Svalbard was included in the UNN Tromsø´s catchment area. The following four sources were employed:

- The Norwegian Patient Registry (NPR). The period 2012–2014 was used when analysing data for the whole country and 2013–2015 when performing further sub-analysis of the northern region. This is due to the fact that data on a national level was not available for the period 2013–2015.
- The National Quality of Care Database (NQCD), 2012–2014.
- Norwegian Myocardial Infarction Registry (NMIR), 2015.
- The database at the National Air Ambulance Services of Norway (NASN), 2012–2014.

In brief, all patients diagnosed and treated for AMI (ICD-10, I21/I22) at any medical hospital in north Norway during the period were eligible for the study on incidence and treatment. The diagnosis was based on the universal definition of myocardial infarction [18]. The NPR did not distinguish between ST-elevation myocardial infarction (STEMI) and non-STEMI (NSTEMI). Data were extracted from the NPR (PCI = procedure codes FNG02 and FNG05, coronary artery bypass grafting (CABG) = procedure codes FNA00, FNA10, FNA20, FNA96, FNB00, FNB20, FNB96, FNC10, FNC20, FNC30, FNC40, FNC50, FNC60, FNC96, FND10, FND20, FND96, FNE00, FNE10, FNE20 and FNE96) and adjusted for gender and age variations.

The establishing of the NMIR was decided by the Norwegian Parliament in April 2010 and initiated on 1 January 2012. Due to a low level of hospital participation during the initial years, we employed only the 2015 data. The following five quality indicators were included:

- The rate of coverage, defined as the percentage of all AMIs (according to the NPR) that was registered in the NMIR database.
- The percentage of patients (< 80 years) diagnosed with ST-elevated myocardial infarction (STEMI) who underwent any reperfusion (thrombolytic therapy, coronary angiography (CAG) with PCI).
- The percentage of STEMI patients (< 80 years) treated with reperfusion in terms of thrombolytic therapy within 30 minutes or CAG/PCI within 90 minutes after first medical contact.
- The percentage of non-STEMI (NSTEMI) patients (< 80 years) who underwent CAG during therapy.
- The percentage of NSTEMI patients (< 80 years) who underwent CAG within 72 hours after hospitalisation.

Furthermore, we implemented from the NQCD the 30-day survival (first time AMI) figure for the 2012–2014 period. Each hospital’s value was calculated employing the treatment chain method (www.fhi.no/www.helsenorge.no).

Data on air ambulance transportation was accessed from the database at the NASN. All patients, transported by helicopter (rotor wing) or fixed wing aircraft, diagnosed with AMI (ICD-10, I21/I22) were detected and categorised according to urgency (ordered, normal, urgent, acute), gender, time of initiation and the need for an anesthesiologist during transport. This study period was from 1 January 2012 to 31 December 2014.

### Statistical analysis and authorisation

The data from the NMIR was implemented from an open source as aggregated and anonymous data [6]. Concerning the NPR data, the Centre for Clinical Documentation and Evaluation (SKDE) at the NNRHA trust had an approval from the NDI initially for the 2012–2014 period, and later for the 2013–2015 period. They did also have an approval from the Northern Regional Committee for Medical and Health Research Ethics (REK) to publish anonymous data.

The NPR data included the whole cohort and descriptive statistics were employed. Gender and age adjusted rates of AMI, CAG and PCI were calculated and the false discovery rate (FDR) measured. Furthermore, logistic regression, multiple testing and the Chi-square test were used. Significance was set to 5%. When analysing 30-day survival, adjusted mortalities were estimated by logistic regression. The analyses included age, sex, comorbidity and number of prior hospitalisations. The method of Guo-Romano with an indifference interval of 0.02 was used to test whether a hospital was an outlier or not [19]. When comparing sub-groups, institutions and counties with regard to quality of care, we employed the Chi-square test. The study was performed as a quality of care analysis. Consequently, no ethical committee or Data Inspectorate approval was necessary. Similarly, no approval from the Norwegian Social Science Data Services (NSD) was required.

## Results

### Incidence

During the time 2012–2014, NPR registered 50,322 cases of AMI among 42,356 patients in Norway. Details are given in Table 1. The northern region had a 9% higher incidence rate of AMI, compared to Norway in general. Within northern Norway, the northeastern county (Finnmark) had the highest AMI rate per year (4.84/1000 inhabitants) and the county hosting the PCI-centre (Troms) the lowest one (2.8/1,000 inhabitants). The national rate was 3.28/1000 inhabitants.

**Table 1.** Number of patients with acute myocardial infarction (AMI) according to Norwegian region and County in northern Norway in 2012–2014. All rates were adjusted for differences in age and sex. Data from the Norwegian Patient Registry.

	Inhabitants	AMI/1000 inhabitants/year	AMI per year	RR
Norway	5,107,711	3.28	16,774	1.00
Southeatern region	2,854,687	3.25	9,259	0.99
Western region	1,073,220	3.18	3,254	0.97
Central region	702,992	3.33	2,454	1.02
Northern Region	477,812	3.59	1,811	1.09
Nordland county	240,723	3.72	1,000	1.13
Troms county	161,974	2.8	454	0.85
Finnmark county	75,115	4.84	357	1.48

Looking at the incidence of AMI (according to hospital catchment areas), we revealed UNN Tromsø had a statistically significant lower incidence rate (2.7/1000 inhabitants, p<0.0001) and a corresponding higher rate was observed for Hammerfest hospital’s catchment area (5.9/1000 inhabitants, p<0.0001). Furthermore, there were significant variations in the rate of CAG between hospital catchment areas. Details are given in Table 2.

**Table 2.** Rate of acute myocardial infarction (AMI), coronary angiography (CAG) and percutaneous coronary intervention (PCI) and 30-day survival in hospital catchment areas in Northern Norway 2013–2015.

Catchment area	Inhabitants	AMI rate (FDR)	n	CAG rate (FDR)	n	30-day survival % (FDR)	PCI rate (FDR)	N
Kirkenes	26,718	3.5 (0.9348)	94	7.2 (0.1507)	196	88.7 (0.2885)	2.8 (0.7554)	77
Hammerfest	48,806	5.9 (<0.0001)	263	11.0 (<0.0001)	517	89.7 (0.5000)	3.9 (<0.0001)	183
Tromsø	126,809	2.7 (<0.0001)	320	7.4 (0.0002)	890	92.1 (0.0055)	3.0 (0.1199)	363
Harstad	35,541	3.1 (0.2758)	118	5.6 (0.0922)	213	88.4 (0.2161)	2.4 (0.5679)	91
Narvik	27,134	3.1 (0.3768)	96	4.6 (0.0002)	132	86.7 (0.0099)	2.3 (0.4519)	67
Vesterålen	30,431	3.2 (0.4619)	109	6.4 (0.9746)	211	87.5 (0.0259)	2.7 (0.9127)	89
Lofoten	22,832	4.4 (0.0774)	109	6.4 (0.9976)	154	88.7 (0.3155)	2.4 (0.6880)	58
Bodø	83,642	3.7 (0.7270)	307	5.9 (0.0957)	490	88.9 (0.3566)	2.5 (0.7151)	212
Rana	33,693	3.1 (0.2883)	112	4.7 (<0.0001)	162	85.1 (0.0043)	2.1 (0.1746)	74
Mosjøen	16,316	4.0 (0.4453)	74	5.7 (0.2883)	104	— (—)*	2.5 (0.7554)	45
Sandnessjøen	28,367	3.3 (0.5376)	100	7.8 (0.0059)	237	89.9 (0.4705)	3.1 (0.4229)	94
Northern Norway	480,289	3.5	1701	6.8	3305	89.4	2.8	1351

* Figures not given due to <100 AMIs.

### Quality indicators

The 30-day survival following first time AMI revealed inferior results in three hospitals and the statistically significant best result was observed at the UNN Tromsø (housing the PCI-centre) (p=0.0055). Details are given in Table 2.

The 2015 quality of care indicators of the NMIR revealed a rate of coverage ranging between 51% and 99%, a percentage of STEMI patients who underwent reperfusion ranging between 42% and 100% and 0% and 38% of reperfusions were performed within recommended time limits. There was no statistical difference between Northern Norway and Norway in general (p=0.22). Because patients in the southern part of Northern Norway are closer to the PCI centre of central Norway, located at St. Olav’s hospital, Trondheim university hospital, 8.2% of patients underwent PCI in other Norwegian health regions. Furthermore, some patients underwent PCI while they were on holiday or visiting other regions of Norway.

Depending on the primary hospital, the percentage of NSTEMI patients who underwent CAG within 72 hours varied between 30% and 81% and in total 49% and 93% had a CAG performed. Although the university hospital (UNN Tromsø), housing the PCI-centre, had the best quality of care results, we could not document a correlation between quality of care and distance to the PCI-centre. Each hospital’s distance in kilometres to the PCI centre and their percentage of AMI patients’ who had a PCI was plotted. A linear regression analysis was performed (p=0.289). Further details are illustrated in Table 3.

**Table 3.** The result of five quality indicators of the Norwegian Myocardial Infarction Registry (NMIR) for 2015.

	Coverage* (%)	STEMI thrombolysis/ CAG/PCI (%)	STEMI thrombolysis <30 minutes or CAG/PCI <90 minutes (%)	NSTEMI CAG <72 hours (%)	NSTEMI CAG (%)
Norway**	88	94	38	58	75
Northern Norway	92	94	20	57	75
Kirkenes	51	100	0	64	93
Hammerfest	99	42	0	30	54
Tromsø	98	99	21	81	89
Harstad	96	100	7	74	89
Narvik	73	100	0	64	77
Vesterålen	98	86	0	38	66
Lofoten	94	88	17	37	60
Bodø	84	93	35	57	75
Mo i Rana	93	96	17	39	49
Sandnessjøen	96	91	38	43	79
Mosjøen	92	100	0	38	57

* Coverage is the percentage of AMIs registered in the NMIR.

** The total figure of Norway includes also the figure of Northern Norway.

### PCI, CABG and incidence of AMI

The PCI rate was significantly higher in one hospital (Hammerfest) catchment area (p<0.0001). The correlation between the incidence of AMI and revascularisation (PCI, coronary artery bypass grafting [CABG]) within each hospital’s catchment area revealed that revascularisation was significantly (p<0.01) more common in the catchment area of UNN Tromsø. Details are shown in Table 4. There was no correlation between 30-days survival and rates of PCI (p=0.18), revascularisation (p=0.97), AMI or revascularisation/AMI rate (p=0.30). Furthermore, there was no correlation between 30-day survival and rate of STEMI (p=0.64).

**Table 4.** Number of revascularisations, AMI, CAG and the rate of AMI/revascularisation according to hospital catchment area in 2012–2014.

Hospital catchment area	Revascularisations*		AMI (A)	CAG**	Ratio A/T
	PCI	Total (T)			
Kirkenes	81	100	100	201	1.0
Hammerfest	179	200	257	541	1.3
Tromsø	375	464	322	945	0.7
Harstad	91	115	127	219	1.1
Narvik	62	82	121	146	1.5
Vesterålen	84	104	119	210	1.1
Lofoten	66	81	127	167	1.6
Bodø	198	260	333	504	1.3
Mo i Rana	72	90	121	161	1.3
Sandnessjøen	83	108	100	234	0.9
Mosjøen	48	58	83	117	1.4

*Revascularisation = PCI and/or coronary artery bypass grafting (CABG).

**CAG was performed as a diagnostic procedure/control or as a combined CAG-PCI procedure.

### Fixed wing and rotor wing air ambulance services

A total of 2383 AMI patients were transported to hospital using fixed wing and 338 patients by rotor wing. This accounted for a total of 12.3% and 7.3% of the fixed wing and rotor wing activity, respectively. Whereas all rotor wing transportations had an anaesthesiologist on board, this was the case in 18% of fixed wing transportations (41% of acute or urgency operations). A nurse anaesthetist was a member of the fixed wing crew. Urgent and acute operations accounted for 46% of all AMI transports (99% of rotor wing and 39% of fixed wing operations). Details are shown in Table 5. When looking at the time of initiation of the transport, we did not reveal any significant pattern during the day for rotor wing missions. However, when looking at fixed wing, a significant drop was disclosed between 4 and 5 p.m., causing an unequal service during the day. When exploring this finding, we revealed the change of crew was performed almost simultaneously. Consequently, around 4 p.m. most planes were returning to their base for the exchange of crewmembers.

**Table 5.** An overview of the air ambulance activity (fixed wing and rotor wing) in acute myocardial infarction in northern Norway, according to the requisition forms filled out.

		Fixed wing		Rotor wing		
Variable		n	%	n	%	Total
Patients		19,298	80.6	4,637	19.4	23,935
AMI	Total	2,383	87.6	338	12.4	2,721
	Females	737	89.9	83	10.1	820
	Males	1,603	85.9	264	14.1	1,867
Urgency*	Ordered	221	99.5	1	0.5	222
	Normal	1,241	99.8	2	0.2	1,243
	Haste	323	86.1	52	13.9	375
	Acute	598	67.9	283	32.1	881

* The urgency alternatives when requesting an air-ambulance: ordered (no urgency given), normal, haste and acute. There were no definitions given for the alternatives.

## Discussion

We have revealed new knowledge concerning the incidence of AMI in northern Norway.

Trends in the population burden of cardiovascular disease (CVD) and associated lifestyle factors differ between regions of the world [20–23]. Studies of the temporal association of these patterns suggest that changes in lifestyle factors precede the change in CVD outcomes.

Effective cardiovascular treatment may also contribute to a decline in CVD mortality [21].

For decades, the higher incidence of AMI in northern Norway has been well known and especially the higher incidence in Finnmark County [24]. However, the analysis based on hospital catchment area revealed a large difference within Finnmark County (between Hammerfest and Kirkenes hospital). Obviously, this must be followed up. Furthermore, the very low incidence in Troms County was remarkable and should be further elucidated. The city of Tromsø has, since 1994, been the site of the population based “Tromsø study” [25,26]. For more than 20 years (seven rounds), this study has analysed the risk of coronary heart disease within the population. This may have influenced the risk of AMI. Furthermore, the city houses the regional PCI centre and the UiT–The Arctic University of Norway. The superior access to cardiovascular interventions and the high number of academicians living in the city may also have lowered the risk of AMI [27].

We revealed significant variations in 30-day survival rate and access to CAG and revascularisation. It was argued back in the 1990s that primary PCI offered the best treatment results [28]. At that time, patients successfully treated with thrombolytic therapy did not regularly undergo CAG [29]. However, in today’s studies on pharmacological-invasive strategies, in example the STREAM study, no significant difference between pre-hospital thrombolytic therapy and primary PCI has been observed, in patients with limited symptom duration [30]. Due to significant distances and only one PCI centre, most patients cannot undergo primary PCI in our region. Based on the STREAM study, it is understandable that we have achieved the same 30-day survival as observed in the other Norwegian health regions performing mostly primary PCI. This is due to the fact that we have put significant efforts into optimising thrombolytic therapy and that we strive to provide such treatment in the pre-hospital setting. Today our emergency medical services perform the electrocardiograms (ECG) and communicate electronically with the cardiologist/internist at the local hospital for diagnosis. When a STEMI diagnosis is confirmed, thrombolytic therapy is indicated and performed by ambulance workers. In the future, the ambulance personnel may be better educated and even perform the ECG diagnosis and, consequently, the procedure may be speeded up.

The hospital catchment model documented significant variations in 30-day survival in our region. Despite these variations, survival rates in Norway have been documented among the best in Europe and a positive trend has been observed in most hospital catchment areas during the last decade [9,31,32]. In this study, 30-day survival was measured employing the “treatment chain model”. However, local hospitals may be bypassed in the treatment chain, generating differences in case mix and making the treatment chain model difficult to interpret.

In future studies, we recommend the treatment chain model compared with a hospital catchment model.

Several international guidelines recommend that NSTEMI patients should undergo CAG within 72 hours after hospitalisation and some have advocated for a 24-hour limit [33–35]. In 2015, 57% of NSTEMI patients in our region weres treated within 72 hours. This was far from the acceptable level of at least 70% [8]. The delay may be due to in-hospital factors at the local hospital, logistics and causes at the PCI-centre. All three alternatives have to be followed up in the future. At present, we are aware that the proportion is improving. During the last quarter of 2016, 81% of NSTEMI patients underwent CAG within 72 hours (personal communication, Thor Trovik, UNN Tromsø). We strongly believe this is an effect of the national quality of care register.

Today, patients with AMI experience prolonged survival and improved quality-of-life (QoL) [36,37]. The NMIR had no QoL data and such studies were almost absent in the medical literature. Consequently, we could not document any differences in QoL between hospital catchment areas. However, we are aware of plans for implementing QoL measures in both the NMIR and the Swedish register (SWEDEHEART) and results could be available within a few years.

Finally, we observed significant differences in the access of air ambulance resources during the day. A more asymmetric exchange of crewmembers will be implemented.

## Conclusion

Significant variations in the rate of AMI and unequal service within the region was revealed. Initiatives to minimise differences in quality of care must be taken. Quality of care indicators are important instruments in the struggle for improvements in the healthcare of AMI patients. In the future, we have to further optimise the treatment chain and we believe QoL figures will be important for the fine-tuning of treatment strategies in the near future.

## Publication/presentation

The study or its data has not been sent to or published in any medical journal. It was presented as a poster (Q84) at the International Forum on Quality & Safety in Health Care in London, April 2017.
